# A case of fetal myocardial calcification with hyperechoic area in fetal echocardiography

**DOI:** 10.1111/ped.15868

**Published:** 2025-01-22

**Authors:** Takeshi Sato, Yosuke Washio, Hotaka Kawai, Kenji Baba, Eriko Eto, Hisashi Masuyama, Hirokazu Tsukahara

**Affiliations:** ^1^ Department of Pediatrics Okayama University Graduate School of Medicine, Dentistry, and Pharmaceutical Sciences Okayama Japan; ^2^ Department of Oral Pathology and Medicine Okayama University Graduate School of Medicine, Dentistry and Pharmaceutical Sciences Okayama Japan; ^3^ Department of Obstetrics and Gynecology Okayama University Graduate School of Medicine, Dentistry and Pharmaceutical Sciences Okayama Japan

**Keywords:** myocardial calcification

Fetal myocardial calcification is a rare pathological condition characterized by diffuse hyperechoic areas in the heart on fetal ultrasound. It can cause severe myocardial dysfunction and occasionally leads to hydrops fetalis.^1^ Pathological manifestations include extensive fibrosis and calcification of the pericardium and myocardium; however, its pathogenesis has not yet been elucidated.^2^ Familial cases are rare, with only three male siblings reported in the past. Herein, we report a case that represents a familial occurrence in a female sibling.

A 35‐year‐old woman (gravida 3, para 0) with a spontaneously conceived singleton pregnancy was referred to Okayama University at 18 weeks gestation due to the appearance of hyperechoic areas in the fetal right ventricular wall on ultrasound. Her reproductive history included two previous miscarriages. The first was a spontaneous miscarriage at the 5th week of gestation. The second was an in utero fetal death, occurring at the 17th gestational week. An ultrasound of the latter revealed hydrops and an entire circumferential hyperechoic lesion of the ventricular wall.

Ultrasound showed fetal right ventricular wall hypertrophy and poor contractility at 20 weeks gestation. Hypertrophy extended to the left ventricular wall, and a decrease in contractility was observed at 24 weeks gestation, whereas the cardiothoracic area ratio increased to 48.6%. Additionally, a hypoplastic left ventricle was not observed. We were unable to identify whether the hyperintense areas were present in the endocardial or myocardial layer. Furthermore, fetal subcutaneous edema and ascites were noted (Figure 1a), leading us to suspect congenital cardiac diseases, such as EFE, rhabdomyoma, and idiopathic infantile arterial calcification. At 27 weeks gestation, fetal edema worsened, and the mother showed signs of mirror syndrome, such as edema, anemia, and hypoalbuminemia. Serological analysis was negative for TORCH syndrome and autoantibodies (Table S1). At 27 weeks 6 days gestation, the mother underwent an emergency cesarean section due to exacerbation of maternal edema. She gave birth to a 1456 g female neonate who died within 15 min of delivery. Her Apgar Score was 1/1. Written informed consent was obtained from the parents.

**FIGURE 1:**
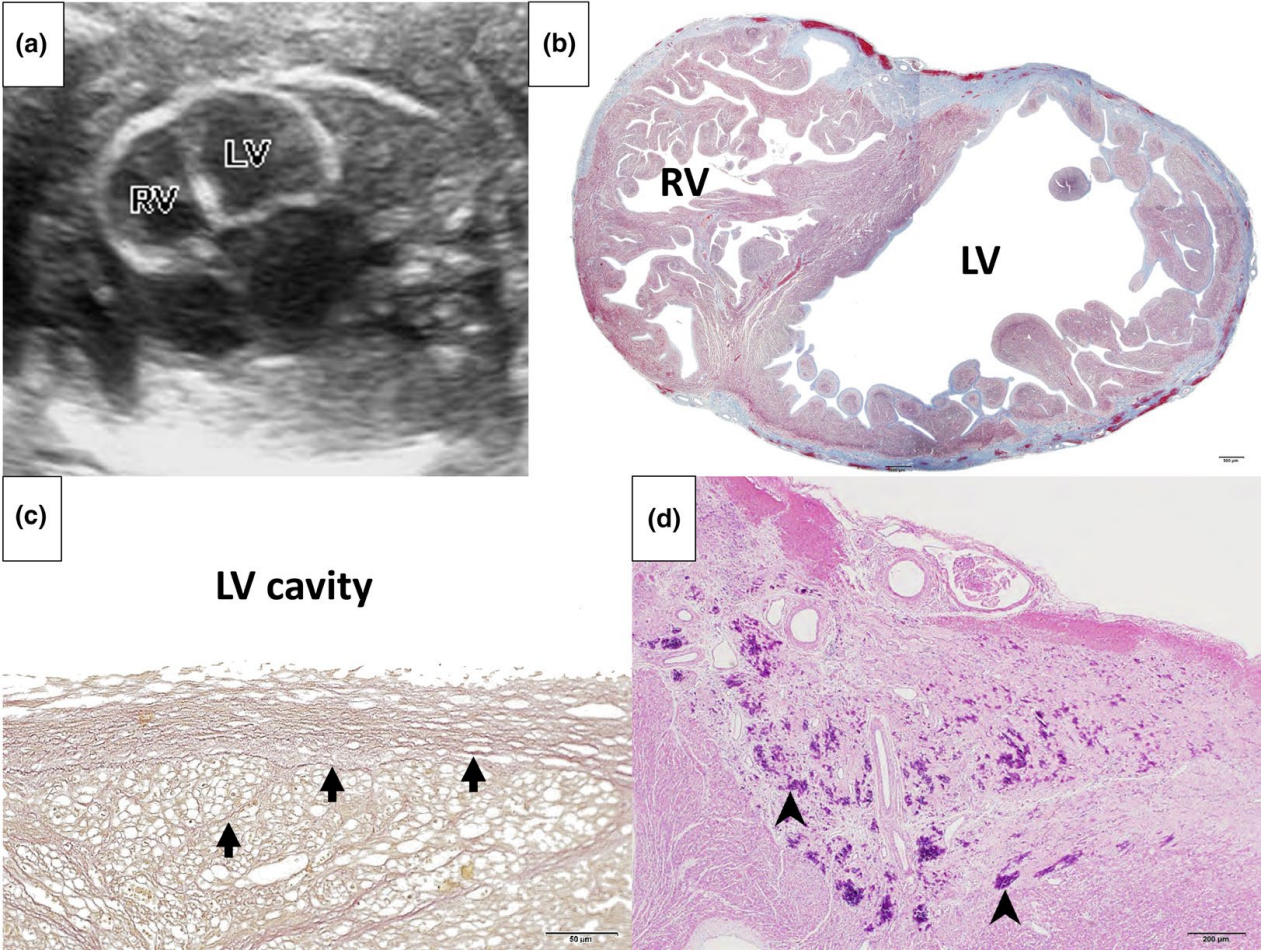
(a) Fetal heart on ultrasound at 24 weeks 3 days gestation. Bilateral ventricular myocardium shows hyperechoic areas and decreased contractility. Cardiothoracic area ratio increased to 48.6%. Hypoplastic left ventricular is not shown. (b) Masson trichrome staining; collagen fiber stains blue. Normal pericardium is extensively replaced by collagen fibers, especially in the left ventricle. (c) Elastica Van Gieson staining; collagen fiber stains red, elastic fiber stains dark purple, and muscle fiber stains yellow. Proliferation of collagen fibers (arrows) is visible. (d) Hematoxylin and Eosin staining; calcified area stains deep purple (arrowheads). LV, left ventricle; RV, right ventricle.

There were no external malformations, and significant symptoms included generalized edema and abdominal distension. The neonate's karyotype was 46XX.

Pathological study of autopsy findings showed cardiac enlargement, hepatomegaly, ascites, and pleural and pericardial effusion.

Histologically, the endocardium was noticeably enlarged from the normal thickness of 10 μm. The endocardial thickness of the left and right ventricles were 70 and 20 μm, respectively.^3^ The bilateral ventricular endocardium demonstrated extensive growth of collagen fibers, without the growth of elastic fibers. Diffuse fibrosis and calcification were also observed in the epicardium (Figure 1b–d). In the left ventricle, the myocardium was almost completely replaced by fibrous components. Based on these pathological findings, we diagnosed this case as fetal myocardial calcification.

Fetal myocardial calcification is a rare condition characterized by the appearance of diffuse hyperechoic areas in the myocardium on ultrasound.^1^ Pathological changes include fibrosis and calcification of the pericardium and myocardium. The cause of myocardial calcification has not been determined, but the pathological condition is considered to be a terminal result of reaction processes such as hypoxic–ischemic injury, infection, inflammation, or drug abuse.^2^, ^4^ Several reports link myocardial calcification with comorbid disorders, including large cardiac malformations, maternal immunologic disorders, and chromosomal abnormalities.^1^, ^2^ In our case, TORCH syndrome and maternal autoimmune diseases were ruled out, and no chromosomal abnormalities were found.

In this particular case, a distinguishing factor was that the fetus of the previous pregnancy showed clinical findings suggestive of fetal myocardial calcification, and there was a high possibility of consanguinity. Although solitary cases are more common, Haug et al.^5^ reported fetal myocardial calcification in three male siblings, suggesting the probability of familial fetal cardiomyopathy. We have identified that familial fetal myocardial calcifications can potentially occur in a female sibling. Extensive genetic analysis was not performed because parental consent could not be obtained; therefore, the genetic anomaly inducing this symptom remains unclear.

As myocardial calcification is diagnosed pathologically, performing an autopsy is important when suspected. Moreover, given that fetal myocardial calcification can potentially occur in family siblings regardless of sex, if a fetus is diagnosed with myocardial calcification in utero, close follow‐up with ultrasound should be performed in subsequent pregnancies.

## AUTHOR CONTRIBUTIONS

T.S. conceived and designed the study, and wrote the manuscript; Y.W. collected the data, and critically revised the manuscript; H.K. and E.E. contributed to data collection; K.B., H.M. and H.T. critically revised the manuscript. All authors have read and approved the final manuscript.

## CONFLICT OF INTEREST STATEMENT

The authors declare no conflict of interest.

## Supporting information


Table S1.
