# Supplementary material for: A case of fetal myocardial calcification with hyperechoic area in fetal echocardiography
Source: Pediatr Int. 2025 Jan 22;67(1):e15868. doi: 10.1111/ped.15868 (PMC11752414; doi:10.1111/ped.15868)
Supplement: Supplementary file 1 — Table S1. [file PED-67-e15868-s001.pdf]

Table S1. Mother's Serologic laboratory test results at 24 weeks 3 days gestation

Infectious agents		Units
STS	<0.7	R. U.
TP antibody	<5.0	T. U.
CMV IgG	40	U/mL
CMV IgM	<0.70	TV
Rubella IgG	<10	IU/mL
Rubella IgM	<0.8	TV
VZV IgG (EIA)	24.1	
VZV IgM (EIA)	0.37	
HSV IgG (EIA)	71.4	
HSV IgM (EIA)	0.26	
PVB19 IgM	(-)	
Toxoplasma IgG (EIA)	(-)	
Toxoplasma IgM (EIA)	(-)	
HBsAg	<1.0	C. O. I
HBsAb	<1.0	mIU/mL
HBcAb	<1.0	C. O. I
HCV	<1.0	C. O. I
HIV	<1.0	C. O. I
**Autoantibodies**		
ANA	<0.08	Ratio
Anti SS-A antibody	<0.50	U/mL
Anti SS-B antibody	<0.50	U/mL

Abbreviations： ANA, antinuclear antibody; STS, serologic test for syphilis; TP, treponema pallidum; CMV, cytomegalovirus; VZV, varicella zoster virus; EIA, enzyme immunoassay; PVB19, parvovirus B19; HBsAg, hepatitis B surface antigen; HBsAb, hepatitis B surface antibody; HBcAb, hepatitis B core antibody; HCV, hepatitis C virus; SS, sjogren syndrome; C.O.I, cut off index
